# Collective insights of public-private partnership impacts and sustainability: A qualitative analysis

**DOI:** 10.1371/journal.pone.0254495

**Published:** 2021-07-20

**Authors:** Sheryl Strasser, Christine Stauber, Ritu Shrivastava, Patricia Riley, Karen O’Quin

**Affiliations:** 1 Department of Health Policy and Behavioral Sciences, School of Public Health, Georgia State University, Atlanta, Georgia, United States of America; 2 Department of Population Health Sciences, School of Public Health, Georgia State University, Atlanta, Georgia, United States of America; 3 Division of Global HIV and Tuberculosis, Centers for Disease Control and Prevention, Atlanta, Georgia, United States of America; 4 Department of Sustainable Development, Region Värmland, Karlstad, Sweden; University of Lincoln, UNITED KINGDOM

## Abstract

The global Coronavirus or COVID-19 pandemic exposed the weakness of healthcare systems including laboratory systems and is a call to action for unprecedented collaboration and partnerships to deal with the global crisis. The United States (U.S.) President’s Emergency Plan for AIDS Relief (PEPFAR) establishes the global HIV/AIDS treatment agenda in alignment with the UNAIDS 90-90-90 treatment targets to achieve epidemic control related to enhanced testing, treatment, and viral suppression. A strategic PEPFAR priority area recognizes that large-scale collective efforts and sharing of resources bear greater potential impact for lasting change than any single organization or entity can achieve alone. An important vehicle utilized within the global public health context is the public-private partnership (PPP) model whereby multiple international organizations forge unified project charters to collectively reach mutually agreed goals. While touted as an ideal mechanism to synthesize resources and maximize gain in numerous applications, little is known from a seasoned stakeholder perspective regarding PPP implementation and sustainability issues. The purpose of this research is to holistically examine perceptions of PPP model sustainability related to inputs and impacts among a collective network of stakeholders experienced with PEPFAR workforce development, laboratory-system strengthening project implementation. Interviews were conducted with frontline stakeholders from public and private sector organizations based in the US and select PEPFAR-supported priority countries. Analysis revealed three dominant themes: PPP impacts, keys of successful collaboration, and logistical challenges and opportunities to enhance sustainability of PPP outcomes in the future.

## Introduction

Public-private partnerships, or PPPs is a term that can be used to describe a broad category of activities and structures involving public and private sectors, defined by the World Health Organization [WHO] broadly as: “wide variety of ventures involving a diversity of arrangements, varying with regard to participants, legal status, governance, management, policy-setting prerogatives” [1]. At minimum, a PPP involves one core public, non-commercial organization and one private or commercial organization that join to share efforts and rewards of the collaboration [2]. It is generally agreed that PPPs have or should have social value, or contribute to improved health of vulnerable populations, generally in terms of increased healthcare system efficiency and access, decreased costs, and increased innovation or creativity [3]. The results of a 2014 systematic review revealed there is a strong focus within the extant PPP literature on efficiencies attributed to graduated models of public healthcare infrastructure and delivery support delegated to the private sector [4]. The most common PPPs involve traditional procurement contracts to support physical infrastructure or building maintenance [5]. However, there is increased interest in initiation of PPPs that establish new PPP goals such as public health intervention scale up and health systems capacity building [6]. The characteristics that drive innovative hybrid PPPs, such as bypassing bureaucratic public contracts and governance, raise crucial questions as to how such PPPs pose both benefits and limits by design. Likewise, they require expanding outcome evaluative parameters to include considerations related to PPP inputs and sustainable development [7, 8].

Metrics that quantify PPP-strategy outcomes beyond finance and health indicators are often neglected in public health research. Using a value-based partnering lens can shift focus towards the quality of relationships and network partner interactions which can enumerate benefits beyond disease transmission trends and viral suppression rates [9]. Reflecting on synergies and compromises made during PPP implementation has the potential to reveal strategic avenues to create and nurture long-term impactful relationships and individual organizational engagement [10]. Retrospective evaluations addressing global health PPP sustainability are rare, yet they provide the ability to explore both expected as well as unintended impacts, which are critically important when exploring lasting impacts of partnerships [11, 12].

## Background

The commitments, orchestration, and perseverance of time, resources, and expertise required to achieve the ambitious United Nations Program on HIV/AIDS (UNAIDS) goals and to meet global 90–90–90 targets for epidemic control are highly complex. The United States President’s Emergency Plan for AIDS Relief (PEPFAR) operationalizes the implementation approach guided by the Office of the U.S. Global AIDS Coordinator’s (OGAC’s) priorities that ultimately work towards the UNAIDS 90-90-90 targets [13]. A critical recognition in PEPFAR 3.0 is that public support and resources required for this effort exceed the capacity of any individual governmental organization and/or international partner to bolster system-level capacity to effectively maintain HIV/AIDS disease control and prevention in low-income settings [14]. Therefore, there is an increasing presence of PPP projects dedicated to global health system improvements involving resource sharing from a broad representation of government and private sector organizations [15–17]. Touted as one the many benefits of PPPs is offering latitude to partners on how to operationalize projects, as they differ from traditional service delivery contract models in terms of flexibility and have a less apparent risk and reward structure [5].

The aim of the UNAIDS Scale Up Goal agenda was to diagnose 90% of all HIV-positive persons, provide antiretroviral therapy (ART) for 90% of those diagnosed, and achieve viral suppression for 90% of those treated by 2020 [18]. Despite significant progress towards controlling the HIV pandemic, at the halfway point, the UNAIDS executive team delivered a ‘wake-up call’ to for those dedicated to global HIV response, acknowledging that both efforts and funding required to support attainment of the 2020 targets were noticeably waning [19].

The PEPFAR *Roadmap for Shared Responsibility* elevated the spirit of PPPs by emphasizing responsibility: signifying that every sector and every individual organization plays a contributing role to foster vitality of a social ecosystem [20]. PEPFAR defines PPPs as a collaborative endeavor that combines monetary and in-kind resource investments from the public and private sector to address PEPFAR’s HIV/AIDS prevention, care, and treatment goals through active engagement that transcends prescribed contractual obligations. Unlike many procurement-based PPP contracts, the PEPFAR-supported PPPs utilize a Memorandum of Understanding (MOU), executed by public and private authoritative executives, which outlines the collaborative goal and primary partner resource commitments. The Director of Centers for Disease and Control and Prevention (CDC) Africa, Dr. John Nkengasong, described PPPs as a critically important paradigm shift in global infectious disease prevention efforts [21]. Within PEPFAR, there is support from private and public sectors highlighting the valuable role PPPs play when striving to resolve health threats in low-resource settings [22–24].

There are few public health PPPs that focus on investments in the development of personnel who conduct the core lab functions critical to the UNAIDS Scale Up agenda. The strength and capacity of laboratories to provide accurate diagnostic tests for timely case detection, treatment initiation, and maintenance to achieve viral suppression is integral to PEPFAR’s success [25]. However, strategic plans to attain 90-90-90 targets have overwhelmed many public health laboratory systems in sub-Saharan Africa due to increased demand for early infant diagnostic (EID) testing of HIV-exposed infants and viral load (VL) monitoring of patients on antiretroviral therapy [26]. Despite system-level demands, PPPs launched in laboratory settings have become increasingly more attractive in arenas where infrastructure development and healthcare service contracts are losing favor [27].

While PEPFAR 3.0 articulates action agendas in four key areas: impact, efficiency, partnerships, and sustainability (described as operating “in lock step with partner countries as they assume greater responsibility for controlling their own country’s epidemic”) [14]; research dedicated to how multi-sectoral collaborative public health networks function in ways that are impactful, optimize partner experiences, and contribute to long-term system improvements and future expansion is scant [2]. Partnerships are recognized as vital in creating sustainable initiatives that are built for long-term durability yet researchers have called for a more profound exploration of how inputs and implementation processes on the partner-level relate to program impacts deemed significant to individual partners as well as the collaborative itself [10]. There are some studies that have begun examining PPP implementation processes. In a comparative analysis examining factors of PPP initiation, Koppenjan identified patterns associated with positive initiation of PPPs concerning transportation infrastructure enhancements [28], and in another, researchers probed insights into the process of implementation by interviewing core actors involved in three Dutch transport PPPs, asking questions relative to project outcomes [29]. However, these studies pertain specifically to infrastructure maintenance PPPs and a need to examine the implementation processes and impacts of PPPs within public health settings remains.

Research exploring PPPs often focus on comparing partner characteristics, values, and tensions that occur in the midst of quantifying collaborative success. Relatively unchartered territory is the investigation into PPP outcomes reported by diverse collaborators based on their vantage point of the dynamic implementation experience [8]. In this work, we examine recollections of the PPP experience among a diverse network of laboratory-based implementing partners using a Theory of Sustainability lens [30], with the aim of answering the following evaluation questions: 1) how do planned resource and input expectations relate to observed project outcomes? And 2), how do outcomes relate with stakeholder perspectives regarding PPP model sustainability? The evaluation approach is based on principles of Participatory Action Research which centers on reflecting on learned knowledge in order to influence practice [31]. Key stakeholders were interviewed about their collaborative experience as a means to better understand interactions that served a purpose, endured, failed, or perhaps changed. Themes from the responses present rich information for global health leaders to understand a more comprehensive concept of effective PPP implementation practices and effective alliances that can support lasting change [32]. The case network design allows for a collective analysis of themes that emerge across network partners aiming to advance a vast global health intervention agenda within the context of public health laboratory systems strengthening.

### PPP case network

In 2007, OGAC, the U.S. CDC, and Becton Dickinson and Company (BD) launched its first PEPFAR supported PPP [33]. Since then, two additional PPPs have been established: one with Roche in 2013 [34], and most recently with Siemens Healthineers in 2014 [35]. The PPPs are linked by a charge to strengthen laboratory systems and enhance workforce capacity to accelerate progress towards attaining the 90-90-90 targets accomplished through diverse training, mentoring, and accreditation efforts. Unified by a common goal, each laboratory-based PPP in the network was distinct. As shown in Table 1, PPPs varied in terms of: the adopted 90-90-90 target, training approaches implemented, modalities employed to engage laboratorians and partners, in addition to project scope, longevity, and level of committed resources.

**Table 1:** Summary of three lab-based PPPs by history, scope, and PPP focus.

Private Partners	Siemens-Healthineers	Becton Dickinson & Company (BD)	Roche Diagnostics
**Year of MOU**^**a**^	2014–2019	2007–2012 (Phase 1)	2012–2017
		2012–2017 (Phase 2)	
**Shared Resources**^**b**^	$15 M USD	$18 M USD (Phase 1)	$10 M USD
		$20 M USD (Phase 2)	
**Goals**	Build a competent laboratory workforce	Improve access & coverage to treatment via stronger specimen referral & timely reports of accurate results	Educate and train laboratory scientists via didactic and online courses to strengthen in-country laboratory networks and systems
**UNAID Target**	First 90	Second 90	Third 90
**Challenge(s) Addressed**	1) Inadequate numbers competent workforce in both laboratory and non-laboratory settings to provide quality assured HIV test results.	1) Non-standardized specimen referral & result reporting system leading to reduced access and coverage of HIV treatment services	1) Lack of understanding among clinical laboratory professionals to perform systematic investigations towards the root cause for common Quality Control scenarios & propose effective corrective actions.
	2) Accelerating global laboratory workforce competency to control HIV epidemic and beyond via e-platform at minimal cost to PEPFAR.	2) Poorly performed phlebotomy compromises patient safety, healthcare worker safety, specimen integrity.	2) Inadequate reach & coverage of standardized refresher training material for VL/EID^c^ specimen collection
			3) Low percentage of VL/EID^c^ results uptake from laboratory in the clinics.
**Mode of training**	E platform- PEPConnect [36]	Expert volunteer Global Health Fellow mentors sent in country [37]	Didactic training offered at Roche Scientific Center [38, 39] Johannesburg and via web portal of African Society of Laboratory Medicine
**Countries served**	Pilot in Uganda with potential for global access	Ethiopia, Uganda, Kenya, Mozambique, South Africa, India	Online tools with ability for global access
**Topics of focus in training activities**	1) Rapid testing & Continuous Quality Improvement	1) Support MOH^d^ to strengthen national network of specimen referral and result reporting system	1) Quality Control and Method Validation in clinical laboratories
	2) Quality Control & Method Validation [40]		2) Specimen collection using dried blood spots for VL/EID^c^
		2) Strengthen national laboratory system to develop improved Quality Management System.	3) Improving laboratory clinic interface for improved result reporting & action to treat patients.

^a^ MOU = Memorandum Of Understanding

^b^ M USD = Millions in United States Dollars

^c^ VL/EID = Viral load and Early Infant Diagnosis testing

^d^ MOH = Ministry of Health

## Methods

The methodology of this study was guided by the systematic CDC framework for program evaluation, and foundational guidance for evaluating partnerships [41, 42]. Admittedly, the methodology may present trade-offs in terms of generalizability, yet it serves to optimize the relevance, meaning, and overall utility of findings. Investigative team members from CDC played an integral role in operationalizing the evaluation protocol, selecting the instrumentation, and providing the eligible stakeholder sampling frame. The roles of the external evaluation team members were to secure ethical clearance for the interviews, perform subject recruitment, data collection, coding, analysis, and reporting activities independent of the lead CDC research collaborators. Together, the larger investigative team reviewed the coding themes for more nuanced interpretation of study results and implications for future PPP plan guidance.

### Instrumentation

The design, methods, and instrument used for this qualitative analysis were derived from components of the Public-Private Partnerships in PEPFAR Countries Project (P4) evaluation toolkit. The toolkit was developed by Cardno in partnership with the BD Labs for Life PPP and contains resources and templates that align with the U.S. government, Department of Health and Human Services, CDC, and OGAC regulations, policies, and award management requirements for PPP program administration, monitoring, and evaluation oversight [43]. While the comprehensive toolkit provides resources for both quantitative and qualitative assessment of PPP operations, only the Lab Strengthening Partner Assessment Tool (PAT) was used in this study. The PAT was designed to capture primary qualitative data from key PPP stakeholders representing: the CDC, Ministry of Health staff, health facility staff, and representatives from multi-sector organizations with the goal of understanding the sustainability of PPP impacts [44]. PEPFAR defines sustainability as “the capacity to maintain program services after financial, managerial, and technical assistance from the U.S. and other external donor essentially ceases” [45]. The PAT consists of 4 primary questions with additional probing items (S1 File). Psychometric properties of the instrument are unknown.

### Sample

The eligible stakeholder sampling pool was identified by CDC and included stakeholders involved in PPP program operations, which includes management, program staff, partners, funding agencies and coalition members [46]. This group bears PPP program implementation experience that has been recognized as an important, yet limited source of PPP evaluation data [1, 47]. Professionals identified by the CDC International Laboratory Branch as key stakeholders of the lab-based PPP network carried out global, administrative, or program level design or implementation roles within the three PEPFAR-laboratory strengthening PPPs. Table 2 provides a descriptive summary of stakeholders included in the sampling frame.

**Table 2:** Key stakeholder practice settings and groups recruited for interviews.

Operational Level	Targeted Stakeholder Groups
Global	■ Representatives from Office of Global AIDS Coordinator
Administrative and Program Design	■ Mid-level managers
	■ Representatives from PPP program/team staff
	■ CDC Atlanta representatives
	■ Non-governmental organizations supporting PPP activities on global and/or country level
	■ Technological and subject matter expert consultants
Country and Program Implementation	■ Ministry of Health representatives
	■ CDC in-country lab directors, advisors and team members
	■ Other PEPFAR partners-country program coordinators & liaisons-for private & public partners
	■ Managers of participating laboratories

Semi-structured interviews were conducted in-person and via telephone from April through June 2018. Respondents did not receive incentives for participation. Interviewers obtained verbal consent before beginning an interview. To maintain confidentiality of respondent information, investigators provided assurance that results would be reported in aggregate form and not stratified by partner organization, setting, or operational level.

### Ethical considerations

Evaluators engaged PPP leads and staff (from both the private and public sectors), consultants, and CDC team members to gain an understanding of the development, approaches, and design and delivery aspects for each PPP. These stakeholder discussions informed protocol and data collection tool development. The evaluation and data collection protocol supporting this work were formally reviewed and approved by the Georgia State University Institutional Review Board. The protocol was also reviewed and approved in accordance with CDC human research protection procedures and obtained global health protocol clearance. To maintain confidentiality, the audio files, transcripts, and study files were coded using unique IDs and any identifiable information redacted from transcripts. All computers were password-protected and further safeguarded through a secure server.

Given the potential barrier of open discourse between the evaluation team and network of collaborating partners across and between PPPs and stakeholder organizations, the formal evaluation research protocol data collection, analysis, and reporting of results were conducted in a manner that would protect confidentiality of individual participants and their respective organizational affiliation. To mitigate hesitancy of honest dialogue, the interviewer emphasized the evaluation goal of this effort was not to evaluate any specific company or collaborating PPP partner, rather, the purpose of the interview was to elicit collective insights of PPP effectiveness and sustainability. Interviews ranged from 30 to 50 minutes in length and were recorded using a digital audio recorder. Audio files were transcribed verbatim by the evaluation team and used to conduct the analysis.

## Analysis

Interview transcripts were reviewed to identify and characterize emergent themes corresponding to PPP inputs and outcomes related to sustainability using applied thematic analysis [48]. Key concepts were identified as initial codes. Coding definitions were deductively operationalized through an open coding process to ensure that each code was distinct and then systematically applied to transcripts. Each transcript was independently coded by two evaluation team members and themes were established by combining codes and identifying emerging patterns. Inter-rater reliability was measured by averaging Cohen’s kappa across codes as well as the average percent agreement. Inter-rater reliability was calculated using ReCal2 software [49]. The following primary techniques were incorporated into the analyses to enhance trustworthiness of the findings: (a) minimum of two investigators involved in analysis; (b) triangulation of themes within and across cases [50]; (c) partner engagement in evaluation design; (d) in-depth thematic descriptions; and (e) clarification of preliminary analysis report with CDC partners [51].

## Results

In total, 23 in-depth, semi-structured interviews with key stakeholders were conducted by the evaluation research team. All three lab-based PPPs were represented by stakeholder participants in the study sample. The average Cohen’s kappa to measure concordance was 0.90 with an average of 96% agreement for all codes. Given the goal of this evaluation research study was to explore broad implementation experience related to PPP impacts and issues of sustainability among a diverse network of PPP partners, results are not stratified by partner setting or type. The stakeholder interview guide covered items probing PPP implementation experience and perceptions of impact on laboratorian trainees and system capacity to attain milestones of the HIV/AIDS intervention scaling agenda and potential for sustainability and replication.

Three dominant thematic categories were identified by the evaluation research team regarding stakeholder perceptions of PPPs as a mechanism to advance PEPFAR’s agenda of lab system strengthening and workforce development: 1) perceived impacts of PPPs, 2) keys to success, and 3) challenges and proposed solutions. Results of the thematic analysis and emergent themes and sub-themes are presented in Table 3.

**Table 3:** Thematic analysis presented by coding categories.

Theme	Categories
***I. Impacts of PPPs***	Increased rates of laboratory accreditation
	Improved laboratory efficiency
	Improved communication between doctors and laboratories
	Improved public health system responses
	Improved workforce capacity
	Improved maintenance of supplies and equipment
***II. Keys to Success***	Local capacity building
	Stakeholder buy-in
	Local government engagement
	Leverage existing resources and partner knowledge
	Common goals and priorities, set early and evaluated often
	Identifying local needs and goals that address those needs
	Executive and management buy-in (PPP formation and evaluation process)
	Strategic processes (goal setting, planning); monitoring and evaluation
	High quality mentorship (culturally and linguistically appropriate)
	Financial/budget restraints versus more flexible use of financial resources
***III. with Proposed Solutions***	Business model/global healthcare market changes solved by flexible plans
	Misalignment of partner goals versus clearly defined goals and priorities
	Misaligned mentorship versus suitable, linguistically-appropriate mentors
	Limited interaction versus transparent, consistent communication loops
	Limited strategic planning versus a more inclusive planning process

### Theme 1. Perceived impacts of PPPs

All of the stakeholders interviewed described PPPs as having value, and many outlined positive, and in some cases profound, system-level impacts within the host countries. Several stakeholders indicated that participation in the PPP’s resulted in increased rates of laboratory accreditation: *I see a lot of improvement in the facilities most of these Labs were at zero stars (star zero rating is the lowest) right now we’re talking about five stars*. Others indicated improved laboratory efficiency and improved communication between doctors and laboratories. There was also the perception of improved workforce capacity, with trained staff *able to manage patients more effectively* and improved maintenance of the supplies and equipment *it is building labs to be more nimble in any situation*. Additional perceived impacts included an improved public health system response that went beyond the impact on HIV response systems, for example:

*…Through these partnerships we are not just strengthening the ability of our countries to address the HIV/AIDS epidemic … lab systems are used across the health system for any disease area…what I perceive as an outcome through these PPPs [is] enabling these countries to strengthen their overall public health ecosystem which enables them to address other public health threats…Ebola is a great example-and in some of the countries affected by Ebola…they actually managed to control the outbreak a lot quicker than countries that were affected by Ebola that were not PEPFAR countries*.

### Theme 2. Keys to success

One of the overarching themes that emerged from the data were that there were various key components to successful PPP implementation. One commonly discussed factor that impacted success was local capacity building in various forms *“…In order to achieve the 90-90-90 [there was a need for] a greater depth of skill and capacity…[the PPP] formed a really good foundation to build upon and contribute it towards having a [greater] impact on the 90-90-90 initiative…*.”. Stakeholders described the importance of building local workforce capacity through improved knowledge, training, and, occasionally, improved pay. Other stakeholders described capacity building in reference to the laboratory, “*The bigger picture…is workforce capacity*. *It is building labs to be more nimble in any situation*. *…Concepts of diagnosis*, *of testing*, *of treatment that can be applied across the board…that served both the HIV epidemic and countries overall really well”*. Laboratory capacity could include skills and competency of personnel, but often it included improving maintenance, standard operating procedures and systems. This form of capacity building also included references to communication between laboratories, patients, and doctors. One interviewee expressed that before the PPP project improvement in enhancing capacity, there was a breakdown between labs and clinicians, “*Before*, *most of the laboratory tests were not (used)… to actually manage a patient…That was a cost we were losing and the report was not being used to manage the patient*”. The following quote discusses how PPP capacity building impacted not only project goals but patient healthcare service and delivery:

*“…to be able to manage patients more effectively and ensuring that patients get the necessary quality uncompromised service and results all the way from the time they first check into a clinic to when they get administered their therapy in monitored post therapeutic implementation.”*

Another frequently identified key to success was the need for stakeholder buy-in, especially that of the local government, of which engagement was deemed a hallmark of PPP success. Respondents linked local government buy-in with progress towards project goals, replicability, and sustainability. One stakeholder described the importance of local government buy-in and ongoing engagement in regard to sustainability as follows:

*“And we see the impact in the benefits so with that being said the ultimate sustainability always in my mind comes in building the capacity of the local government and the governments taking over what we are doing… So as part of our PPP we make sure-that government is always at the table,…is always involved,…is learning and gaining, …just as much. So that they can ultimately take over.”*

Additional keys to successful implementation described by stakeholders included the shifting reliance on local resources inputs “*CDC do(es)n’t have to be there now they can do it-the MOH can run with it”* along with changing partner roles. One respondent described local partners’ outlook after receiving PPP trainings as “*…happy*, *they are excited*. *They feel very valued because…they did not have the capacity to support laboratory equipment…Today they can be able to respond to the need in their hospitals … and now they can do it*.” Others identified the need to determine common priorities early in the PPP process “…*where is that ultimate vision in terms of mutual areas (interests) and I think that’s the most important and the first step*” and operating in alignment with local concerns and priorities. One respondent articulated, “…*creating perceptions and managing perceptions where citizens feel like they can get tests done anywhere because the standards of care is going to be equal across the board whether it’s the private sector whether it’s in the public hospitals*”.

Another key to PPP implementation was maintaining sight of ownership, which involved recognition of both local populations and buy-in among leaders, illustrated in this statement, “*who at the end of the day is going to own these particular systems and we need to get them to buy in*. *So on one hand you have government on the other hand you have the citizenship* …”. Many stakeholders felt that high quality mentors/mentorship that were culturally and linguistically appropriate was a key resource related to successful PPP implementation specifically for “*providing technical assistance to fill in a gap that is identified by a country*” and to expand reach of mentors beyond what traditional resources can support on the local scale. Finally, utilizing a strategic process (i.e. goal setting, strategic planning, monitoring and evaluation) were considered vital to successful implementation.

### Theme 3. Challenges and proposed solutions

Stakeholders identified several key areas where implementation challenges arose. Challenges spanned from financial and budget constraints to dramatic changes in business models and global healthcare markets. One challenge identified by multiple stakeholders was that the priorities of various partners did not always align. A common solution proposed was discussing common goals and priorities clearly among partners early in the process and then to continue to discuss and reevaluate said goals and priorities. Below are quotes from two different stakeholders echoing this notion.

*“So I think if someone came to me and said we’d like to replicate this, how do we do that? I’d say first and foremost you have to have your common vision of what are your overall goals and priorities and where do those goals overlap between partners”*

*“…That would be very helpful to make sure before we even engage we are clear on and what we want to do and the priority areas”*

Mentorship by experts to promote professional development was viewed both as a source of tension but if implemented to accommodate partner needs as a potential solution for system strengthening. One illustrative statement demonstrating this sentiment was:

“*One challenge we face is mentors…*.*that are brought in-country*. *Because we have in-country mentors that are trained to do mentorship*, *the in-country mentors kind of feel unhappy because they expect that the outsiders are going to discuss technical systems not provide general mentorship*.*”*

For many, potential solutions to enhance mentorship efforts emphasized use of local mentorship resources and opportunities. For example, one stakeholder spoke of creating a process so that laboratory champions could be used to stimulate others to ‘*actually go to university to go to pre-service institutions to…get more young people excited about being technical engineers*’.

In addition, interactions of partners were not viewed as always ideal among stakeholders and often this was attributed to a misalignment or miscommunication among partners. The following quote highlights this challenge:

*“Another challenge that I think could be done better is sharing the MOU and knowing what the partners are bringing on board….because it created expectations and when you are setting the goal….eventually we realize(d) that is not what they’re bringing to the table…but you could see where our expectations were different.”*

Solutions for improvement of the quality of PPP collaboration included better communication practices. One stakeholder discussed how to enhance collaboration quality.

*“I think what could make this partnership even stronger is if we establish more consistent communication it would help in moving some of these aspects along so there isn’t a hurry up and wait hurry up and go situation on some of these topics… And for consistency we have great communication when we have it but the consistency is where we might identify room for improvement to ensure things are not falling behind or having to be a rushed matter.”*

Some stakeholders indicated that a dearth of knowledge concerning project inputs: partner roles, project goals, and implementation plans as a challenge. For many, the proposed solution was incorporating a more strategic planning process to establish partners’ roles and responsibilities early in the partnership. Others discussed that a lack of monitoring and evaluation capacity as a challenge, and others, like the one below, explicitly expressed it as such. Often these descriptions were coupled with potential resolutions, such as establishing monitoring and evaluation systems. Other stakeholders referred to this challenge as simply a lack of data collection.

*“One of the areas (that has not) been very mature… strong M&E plans for the PPPs from the beginning so… when it came time to really evaluate in a very concrete way …epidemiologically- or statistically- or programmatically-sound way we were a little bit challenged and…we lost a lot of data because we were not able to compare apples to apples from pre- to post- implementation and we recognize that… I think there was a lot more richness there that we may have lost along the way that would have been captured if we would have had a robust M&E plan from the beginning.”*

Finally, other challenges and suggested solutions revealed in our analyses included: 1) executives and managers being removed/disengaged from the PPP process, with the solution being to ensure continuous leadership engagement throughout the PPP implementation cycle; 2) changes in business models and global healthcare markets meant that partnerships had to maintain flexibility and adaptability; and 3) surging costs, budget constraints and funding instability that may prompt reassessment of the funding agreements, resource commitments and renewal processes.

## Discussion

This innovative evaluation addresses a gap in scientific literature by retrospectively exploring diverse stakeholders’ perspectives of PPP implementation experiences relative to inputs, impacts and sustainability potential. The unique contribution of this qualitative investigation captures in-depth perspectives often neglected in evaluative research related to the vantage point of PPP stakeholders with post-implementation experience, especially within the context of strengthening public health laboratory systems and training the workforce in order to catalyze HIV/AIDS intervention scale up goals [1, 4, 24, 52].

PPPs in global health have achieved remarkable successes in critical areas such as vaccine and essential medicine distribution, healthcare infrastructure and maintenance support in which governments and nongovernmental organizations acting alone have struggled [16, 53]. However, PPPs that specifically target the advancement of laboratory staff and networks, an indispensable component of global health programs, are rare. One overarching theme identified in this study was that stakeholders maintain positive beliefs that PPP project inputs contributed to a wide range of laboratory system improvements. Outcomes included greater operational efficiency, improved communication, and enhanced capacity in terms of the workforce, the laboratory network, and of the public health system. This theme aligns with research supporting that previous PPPs have extended intervention and health care treatment reach, in support of the second 90 UNAIDS’ target, resulting in both health system cost-savings and enhanced specimen processing efficiency [24]. In another study, HIV patients gaining access to a private support program sustained by a PEPFAR-PPP resulted in public resource utilization savings and effective viral suppression among enrollees [53]. While evaluation of quantitative impacts of laboratory-supported PPPs was not the aim of this study, the emerging theme suggest stakeholders felt that PPPs strengthened systems and supported progress towards achievement of the UNAIDS 90-90-90 targets. This theme is similar to a survey study of implementing partners recruited from a network of 15 unique community sustainability-focused multi-sector partnerships who were asked to rate both partner-level and collective-group benefits associated with collaborative engagement. The researchers found that respondents with more favorable ratings of their respective organization’s implementation strategy capacity, exemplified by the ability to plan, organize, balance knowledge and resource contributions, was positively linked with greater partner-level and collaborative-wide benefits, including greater progress made toward overall community-wide sustainability goals [54].

In our study, stakeholders identified distinct capabilities among implementing partners attributed to the public or private sector that contributed to system improvements that persisted post PPP-implementation. For example, the ability to access technical expertise in laboratory technologies and achieve greater operational efficiency was noted with respect to private partner impacts; whereas the longstanding reputation of public health leadership and capability of leveraging a widespread global network for influence were noted as valuable contributions facilitated by public partners. These findings align with attributes of respective public and private partner contributions recognized in relation to project outcomes identified in Roehrich’s systematic review of PPPs [4]. Our results are similar to scientific findings that show an equitable balance of inputs and complementary partner contributions is linked with positive collective impact [54]; however, more nuanced investigation focusing on individual partner-level contributions and impacts, as well as assessing specific implementation strategies activities would yield more insightful PPP formation and performance guidance to optimize sustainability.

Scientific support to advance PPP implementation science must keep pace with the expanding presence of hybrid global public health PPPs. Our study findings offer important identification of collaborative conditions that can threaten effective PPP performance and sustainable improvements. Emergent themes indicate that stakeholders demonstrate a remarkable capacity to reflect on implementation experiences that initially presented as challenges, yet they offered pathways into actions by which coordination of resources, active communication, or additional means were utilized to ultimately overcome such obstacles and in some cases-they were viewed as positive PPP impacts. One example framed as a challenge was cultural incongruence occurring between external mentors being inserted into local laboratories. Research demonstrates that limited interagency relationship-building can have significant effects on collaborative performance [55]. Problem-solving and relationship-building among PPP partners is understudied and our findings demonstrate that it is an area worthy of more attention.

The second overarching theme that emerged from our analysis identified experience of PPP implementation associated with the success of PPPs. Stakeholders’ discussed capacity-building, MOUs, partner knowledge, engagement of leadership and local stakeholders, and mutually recognized goals, as important factors associated with health systems improvement and enhanced laboratory capacity. These findings echo components included in a multi-sector, inter-organizational collaborative framework developed by Quélin and colleagues [56]. The framework emphasizes that rules, norms, and values that exist within any individual partner organization are as of equal importance especially when considering contractual, institutional, and resource/process factors that are forged among organizations engaging in a complex partnership.

What does valuable partnering look like? This is the question PPP researchers such as Barlow, Roehrich, and Wright have asked. Lessons from hybrid PPPs have been recognized as the way to understanding complex project goals that do not map into straightforward performance metrics [6]. In our study, stakeholders reflected on intentional relationship-building building between lead organizations and implementation partners as a critical element associated with PPP impacts. Stakeholders that were more knowledgeable of collaborative partners’ involvement and motivations had greater insights into progress made towards goal attainment than those with an unclear understanding of partner roles, engagement expectations, and limited interactions. Overall, cultivating high quality relationships related to partners and mentors were beneficial in addressing the challenges PPP collaboration presented, which represents the third major theme of our analysis.

Stakeholders recalled specific challenges during PPP implementation that threatened PPP outcomes and sustainability. Examples included times of financial uncertainty, or changes within organizations including personnel or business values, as well as shifts in global markets. This aligns with a study conducted in Nigeria which investigated impacts of PEPFAR funding policy modifications as associated with significant reductions in viral load testing, routine drug monitoring labs, staff employment, and overall interrupted lab services [57]. Our study reveals that misalignment of program strategies and cultural competence of experts interacting with in-country teams posed problems that diminished PPP outcomes and potential for sustainability as reflected in responses. However, stakeholder respondents offered solutions to resolve such difficulties based on their lived implementation experience, which may have paradoxically reframed the original problem to be subsequently positive. This is a phenomenon, identified by Niesten, of how collaborating partners may arrive at their own sense of value in working together [58]. Stakeholders articulated that maintaining open, regular communication and facilitation of a collaborative planning and revision process would enhance PPP collaboration interactions and bolster potential for lasting, local impacts. This finding is similar to the Dutch study of transportation PPPs where lack of interaction was identified as a primary cause of PPP stagnation [29]. Within the realm of global health, research supports that the social value of collaboration can be enhanced through establishment of functional, reliable, and verifiable criteria to initiate, implement, incentivize, and evaluate PPPs [6, 59].

Subsequent research dedicated to PPP formation, conceptualization, and operational norms that preserve and maintain trust, justice, and regard for humanity is imperative [55]. Our results corroborate that while PPPs have clear goals for forging multi-sector, global collaborative partnerships to solve complex population health problems, implementation practices of PPPs must build plans that account for fluid organizational, environmental, and contextual conditions that otherwise may limit project sustainability [4, 56, 60]. The failure to prepare multi-sectoral teams on best practices and collaborative implementation science evidence is linked to negative program outcomes including low workforce morale and the dissolution of partnerships [57].

Finally, the greatest potential to strengthen global health PPPs is to advance evaluation efforts. Defining a mutually agreed upon mixed methods monitoring and evaluation plan that includes key PPP process measures and impacts is imperative for determining the overall health of PPPs and their constituent partners throughout the collaborative cycle. This affords the ability to recognize partner contributions, clarify values, track progress, and institute changes that bear the potential of program implementation disruption [1]. Efficient monitoring of data would allow key stakeholders to understand and quantify the effectiveness of diverse workforce development approaches utilized by respective PPPs, which was not consistently discernible among network stakeholders. Enhanced commitment to standardizing monitoring and evaluation activities would support routine transactions and elevate PPP accountability and transparency practices [27], which were expressed as important to sustained project success. While standardized evaluation approaches to quantify PPP impacts are not widely available; models such as the World Health Organization framework [61] and the P4 Toolkit [44], present pertinent domains to assess complex PPPs within the context of public health. However, these resources require adaptations so they include capture of specific laboratory system strengthening project plans from formation through implementation, as well as strategic plans to address ownership and transitions occurring beyond the PPP life cycle [62–66].

The results of our study highlight the creativity supported by PPPs that join private corporations with public health and government organizations to strengthen overall laboratory capacity and healthcare systems to accelerate the UNAIDS 90-90-90 targets in their countries [67]. However, enhancing laboratory capacity as a system has implications that go beyond disease control [68]. There may be contractual challenges, institutional barriers, and partnering regulations that influence a PPP’s ability to function and operate beyond the original MOU [16, 56]. Recognizing that in-country partners may lack the bandwidth to assume expanded responsibilities that monitoring, evaluation, and reporting activities may introduce if PPP partners are not thoughtful in planning an equitable, supportive plan that is responsive to issues of feasibility and partner capacity [1, 16, 66].

Thus, there is a clear need to understand internal and external structures that maintain the vital indictors of PPPs health, which include respect, justice, and trust among partners so that diverse partner interests, engagement incentives, and expectations are harmonized and conflicts/tension are readily reconciled. Research regarding global health PPP implementation practice improvements would also benefit from advanced assessment into ‘the bridge position’ which essentially refers to role of lead partner in a service-based PPP, and oversight of relationship management and coordinating of interactions among network partners. The extent to which a private partner assumes this role may threaten the vitality of a PPP due to power imbalances, greater potential for improprieties, or a shift in operational values that can upend equitable, balanced collaborative engagement [69]. While these occurrences may not have been disclosed by respondents, framing future studies to capture leadership oversight should be a priority. Subsequent PPP evaluation frameworks must examine performance metrics with more direct role delineation, qualification of implementation activities, and quantification of collaborative advantages/impacts on an organizational-level in order to maximize sustainability [66, 70, 71]. The utility of using a qualitative research approach provided a rare opportunity to explore network perspectives regarding the collective impacts and sustainability of PPP collaborative experiences which provides beneficial insights into the formation of new global health PPP partnerships that remain agile in the dynamic global health arena.

### Limitations

There are limitations to this study. The degree to which the study sample represents perspectives of all eligible stakeholders across the PEPFAR-supported system strengthening laboratory network of PPPs is unknown and should be interpreted with caution. Interviews were conducted with key stakeholders identified by a PPP partner which may have allowed overrepresentation of certain partner types/sectors (such as public or private, or U.S. based versus international) and underrepresent others. Additionally, interviews conducted by an external evaluation team, via phone or in-person, and the use of digital recorders may have impacted the robustness of conversation due to variable stakeholder comfort, understanding of confidentiality, and cultural norms regarding research participation. The assurance of confidentiality and small study sample limited the researchers’ ability to examine themes based on stakeholder differences, which would allow for a more granular exploration of themes by select characteristics of individuals and/or organizations. Further, the thematic interpretation may be prone to research bias. Finally, the non-probable purposive sampling approach used in this study does not yield generalizable results.

### Conclusions

Nothing exposes and emphasizes the weakness of healthcare systems including laboratory systems like the COVID -19 pandemic. In our study, the PPP model for collaboration was overwhelmingly viewed as being critically important by stakeholders who stated that PPPs added value in various ways, such as: ‘filling in gaps’; supporting ‘innovative solutions’; and ultimately contributing to building of “a critical mass or critical pool of (laboratory) technologists who are equipped with the latest knowledge and skills in the area of quality management systems” that can outlast a PPP agreement.

Frontline laboratory-based stakeholders involved in prevention, treatment and suppression activities of the HIV/AIDS epidemic who engage in complex hybrid partnerships bear a wealth of insights regarding the perceived impact and sustainability of innovative multi-sectoral collaborations. Collective stakeholder responses reveal a vast potential for PPPs to advance laboratories’ role in contributing to a dynamic whole patient care system that is primed for effective infectious disease response beyond HIV/AIDS, such as COVID-19 and other emerging population health threats. As PPPs continue to increase in prominence within the public health arena, expanded multidisciplinary evaluative research will play a critical role in understanding how dynamic conditions of the global health security agenda and core partners’ need for strategic inclusion can both be served.

## Supporting information

S1 File Interview questions. (PDF)
