# Supplementary material for: Collective insights of public-private partnership impacts and sustainability: A qualitative analysis
Source: PLoS One. 2021 Jul 20;16(7):e0254495. doi: 10.1371/journal.pone.0254495 (PMC8291689; doi:10.1371/journal.pone.0254495)
Supplement: S1 File — (PDF) [file pone.0254495.s001.pdf]

International Lab Branch PPP Key Stakeholder Interview Instrument

Table 1 presents the four primary questions along with sub-questions used to guide interviews for this study. Interviewers presented key questions to each consenting stakeholder, with additional probes used at the interviewers' discretion.

Figure 1. Key questions and sub-questions for each PPP

**Key Question 1:**<br>What were the resource inputs, activities, and outputs of supported activities?	**Sub-Questions:**<br>■ What was the total value of monetary and in-kind resources each partner contributed to the PPP?<br>■ Have the terms of the partnership been adhered to by the partners?<br>■ To what extent have the partnership's original goals and objectives been met?<br>■ Were all deliverables completed by assigned parties?<br>■ Was this partnership essential to meet the objectives and activities?
**Key Question 2:**<br>To what extent has the partnership contributed to improvement towards achievement of the respective 90-90-90 targeted goal of each respective PPP?	**Sub-Questions:**<br>■ What are the short- and medium-term health outcomes at the country level?<br>■ What is the value added of the partnership in terms of PEPFAR's shared goals and resources leveraged (respective of each PPP)?<br>■ How does the output from the partnership translate into health outcomes?<br>■ What were the monitoring and evaluation arrangements, how did they support tracking of program outputs and outcomes and how can these be strengthened in the future?<br>■ Do PPP partners verify the data reported to it by participating country partners?<br>■ What is the intangible value of the PPP?
**Key Question 3:**<br>Is the PPP replicable?	**Sub-Questions:**<br>■ What are the comparative advantages and disadvantages of each PPP model of system strengthening vs. other models of private sector system strengthening support? What are some areas of refinement?<br>■ What are the opportunities emanating from the partnership for future partnerships with PEPFAR?<br>■ What are the lessons from the PPP that can be adopted by other companies?<br>■ What are the critical lessons learned from the PPP? How can these be transferred for the development of future PPPs related to lab strengthening/PEPFAR goals?<br>■ Are there any opportunities for replicability of critical inputs and activities to date?
**Key Question 4:**<br>Is the PPP sustainable in terms of its activities, model, and impact on laboratory systems?	**Sub-Questions:**<br>■ How sustainable is this particular PPP model?<br>■ How has the PPP contributed to sustainable improvements in lab systems?<br>■ How will activities initiated be sustained at the close of the PPP?
